# The Beat

**Published:** 2008-08

**Authors:** Erin E. Dooley

## Will Beijing Clean Up in Time?

**Figure:**
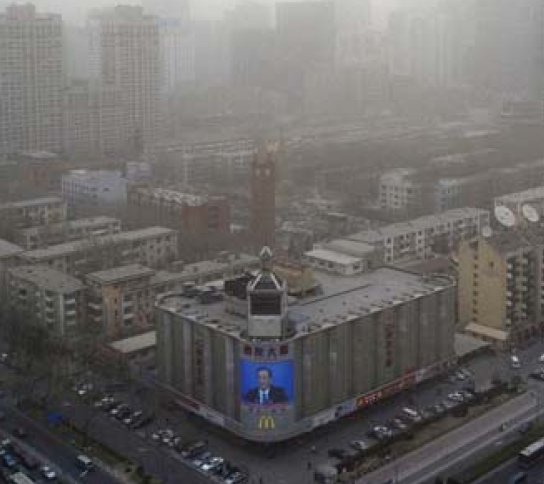
Beijing, 18 March 2008

When Beijing made its bid in 2001 to become an Olympic host city, the city assured the International Olympic Committee that it would meet WHO standards for air pollution by the time the games commenced. As the event gears up for its August 2008 debut, the city has taken drastic measures in a last-ditch effort to reduce pollution levels and improve the air quality. For the past several weeks factories, construction sites, and concrete mixing plants have been shut down, and automobile traffic has been restricted since mid-July. It remains to be seen whether the city’s air will be clean enough in time; Olympic officials warn that outdoor endurance events may be postponed if air quality is poor. The NIEHS has funded research to compare resident lung health before and after the Olympics cleanup.

## Microbes Heat Up

Microbes perform a range of critical functions, such as helping regulate oxygen and greenhouse gases in the atmosphere, fixing nitrogen in soils required for plant growth, and converting waste matter to nutrients. Researchers reported at the June 2008 general meeting of the American Society for Microbiology that climate change is already affecting microbial communities in Alaska, where warmer temperatures are raising nitrogen availability in soils, possibly impacting fungal activity and diversity. Shorter freezing periods could also prevent molds that grow under the snow from retaining enough snowmelt, subjecting trees to drought.

## Rice Not So Nice for Babies?

**Figure:**
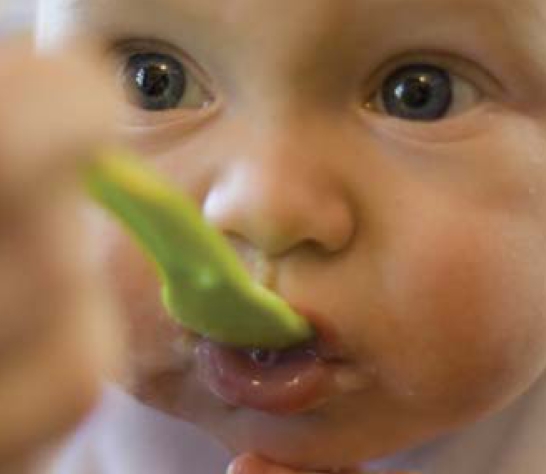


In many areas of the world, babies are weaned from the breast or bottle onto rice cereal and other rice-based foods. A study in volume 152, issue 3 (2008) of *Environmental Pollution* finds that rice foods sold in Western supermarkets can contain high levels of inorganic arsenic—a baby eating 1 serving of rice cereal each day could take in more of this carcinogen per kilogram body weight than an adult exposed to the maximum allowance in drinking water. Arsenic levels in rice vary depending on where it is grown; the authors suggest using rice from lsources in India, California, and Spain.

## Lead in Artificial Turf

Environmental health advocates have recently begun to question the safety of artificial turf fields [see “Synthetic Turf: Health Debate Takes Root,” *EHP* 116:A116–122 (2008)]. Now, after testing in New Jersey revealed high levels of lead on some artificial turf fields, the CDC has recommended that worn playing fields be tested for lead. Moreover, children under age 6 years should not be allowed to play on fields found to have lead levels exceeding 400 ppm. On 23 June 2008, the nonprofit Center for Environmental Health filed suit under California’s Proposition 65, calling for 15 companies to cease producing and selling artificial turf. Three days later the San Diego County Water Authority suspended an incentive program that offered a water bill rebate to consumers who installed artificial turf lawns. The CPSC is investigating lead in artificial turf, with a report expected any day.

**Figure:**
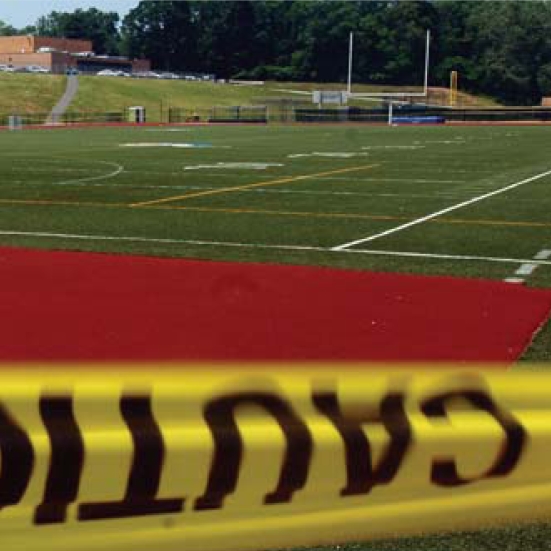
New Jersey football field undergoing testing for lead

## Globalization and Food Safety

The United States is increasingly importing more of its food—approximately 15% in 2006—from developing countries, which researchers at the June 2008 general meeting of the American Society for Microbiology warn have lower sanitary food production standards than those in the United States, especially for seafood and fresh produce. More than 80% of the seafood consumed in the United States is imported, mainly from Asia, where aquaculture practices often involve using raw domestic sewage or livestock manure as feed, as well as the use of pesticides and antibiotics not approved in the United States. The researchers point out that many U.S. companies take steps to verify that their imports have been produced under stringent sanitary conditions. The FDA inspects imported food, although on a limited basis—less than 1% of more than 9 million imports annually.

## Dirt Cheap Lighting for Africa

With only 26% of people in Africa having access to grid-based electricity, many of the continent’s population rely on dangerous and increasingly expensive kerosene lamps and candles for lighting. A new fuel cell system, one of the winners of the World Bank’s May 2008 Lighting Africa competition, may be the solution. The system, developed by Harvard University students—four of whom are from Africa—harnesses energy produced by soil microbes as they break down organic matter such as animal waste and food scraps. The maintenance-free system, which is estimated to be cheaper than solar power, can generate enough energy to power LEDs and radios or to charge cell phones.

**Figure:**
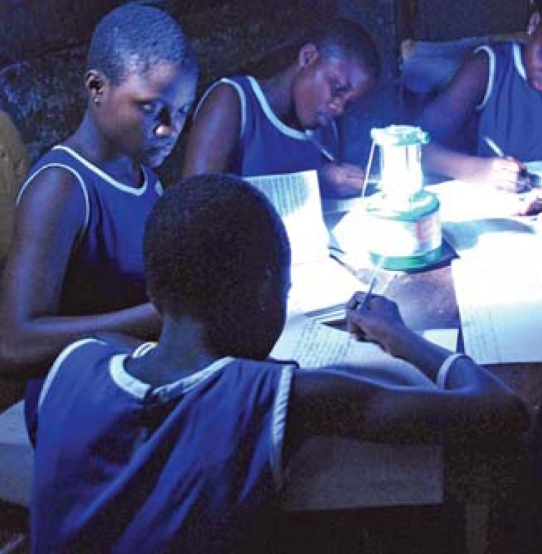
Ghanaian students use LED lighting at home

